# Conference Calendar

**DOI:** 10.1002/cfg.503

**Published:** 2005


					Comparative and Functional Genomics
Comp Funct Genom 2005; 6: 407-409.
Published online in Wiley InterScience (www.interscience.wiley.com). DOI: 10.1002/cfg.503
Conference Calendar
March -- May 2006
Animal models
BSAS -- Annual Meeting, March 27-29
http://www.bsas.org.uk/Meetings
& Workshops/
47th Annual Drosophila Conference, March
29-April 2
http://flybase.bio.indiana.edu/docs/news/
announcements/meetings/
European Worm Meeting, April 29-May 3
http://www.imbb.forth.gr/ewm2006/
Bioinformatics
IBC -- InfoTechPharma, March 14-16
http://www.infotechpharma.com/
10th Research in Computational Molecular
Biology, April 2-5
http://recomb06.dei.unipd.it/
BioIT World, April 3-5
www.bioitworldexpo.com
IASTED -- Modelling and Simulation, May
24-26
http://www.iasted.org/conferences/2006/
Montreal/ms.htm
Functional genomics
KEY -- Malaria: Functional Genomics to
Biology to Medicine, February 28-March 5
http://www.keystonesymposia.org/Meetings/
ViewMeetings.cfm?MeetingID=804
EuroSciCon -- Analysing the Phenotype and
Function of Regulatory T cells, April 4
http://www.regonline.co.uk/eventinfo.asp?
EventId=20 607
EuroSciCon -- Bacteriophage Applications,
May 15-16
http://www.regonline.co.uk/eventinfo.asp?
EventId=24 596
General
ACS -- Spring Meeting & Exposition, March
26-30
http://chemistry.org/meetings/atlanta2006
Experimental Biology, April 1-5
http://www.faseb.org/meetings/eb2006/call/
default.htm
ASBMB -- Centennial Annual Meeting, April
1-5
http://asbmb.interliant.com/asbmb/site.nsf/
main/meetings?opendocument
NSTI -- Bio Nano Conference, May 7-11
http://www.nsti.org/BioNano2006/
CSHL -- The Biology of Genomes, May 10-14
http://meetings.cshl.edu/meetings/genome06.
shtml
GRC -- Chromatin Structure and Function,
May 21-26
http://www.grc.uri.edu/programs/2006/
chromat.htm
Human Genomics and Genetics
GRC -- DNA Damage, Mutation and Cancer,
March 5-10
http://www.grc.uri.edu/programs/2006/dna.htm
CSHL -- Gene Expression and Signaling in the
Immune System, April 26-30
http://meetings.cshl.edu/meetings/immune06.
shtml
Copyright  2006 John Wiley & Sons, Ltd.
408 Conference Calendar
ESHG -- European Human Genetics
Conference, May 6-9
http://www.eshg.org/
HUGO -- Human Genome Meeting, May
31-June 3
http://hgm2006.hugo-international.org/
Metabolomics
KEY -- Metabolomics: From Bioenergetics to
Apoptosis, April 2-7
http://www.keystonesymposia.org/Meetings/
ViewMeetings.cfm?MeetingID=782
4th International Congress on Plant
Metabolomics, April 7-10
http://www.pmet06.org/
Microbes
European Fission Yeast Meeting, March 16-18
http://www.wellcome.ac.uk/doc wtx024 117.html
SGM -- 158th Meeting, April 3-6
http://www.sgm.ac.uk/meetings/MTGPAGES/
Warwick06.cfm
GRC -- Biology of Spirochetes, April 23-28
http://www.grc.uri.edu/programs/2006/spiro.
htm
ASM -- 106th General Meeting, May 21-25
http://www.asm.org/Meetings/index.asp
Pharmacogenomics/Genomics and
Disease
CHI -- Translational Medicine: Bridging the gap
Between Discovery and Development,
February 28-March 1
http://www.healthtech.com/2006/ddd/index.asp
GRC -- New Antibacterial Discovery &
Development: March 5-10
http://www.grc.uri.edu/programs/2006/
antibact.htm
IBC -- 10th Anniversary: Drug Discovery
Technology Europe, March 13-16
http://www.drugdisc.com/europe/
CHI -- Glycomics and Carbohydrates:
Overcoming Challenges in Drug and Product
Development, March 23-24
http://www.healthtech.com/2006/gly/index.asp
KEY -- Structure Based Drug Discovery, April
4-9
http://www.keystonesymposia.org/Meetings/
ViewMeetings.cfm?MeetingID=771
CHI -- Microarrays in Medicine, May 4-5
http://www.healthtech.com/2005/mar/index.asp
KEY -- Genomic Biomarkers: Impact on Drug
Discovery and Clinical Practice, May 7-11
http://www.keystonesymposia.org/Meetings/
ViewMeetings.cfm?MeetingID=770
KEY -- The Molecular and Integrative Basis for
Toxic Responses, May 7-11
http://www.keystonesymposia.org/Meetings/
ViewMeetings.cfm?MeetingID=769
CHI -- Biomarker World Congress 2006, May
16-18
http://www.biomarkerseries.com/
Plants
48th Maize Genetics Conference, March 9-12
http://www.maizegdb.org/maize meeting/
KEY -- Plant Responses to Abiotic Stress,
April 8-13
http://www.keystonesymposia.org/Meetings/
ViewMeetings.cfm?MeetingID=792
3rd International Conference on Legume
Genomics & Genetics, April 9-13
http://www.iclgg3.org/
Copyright  2006 John Wiley & Sons, Ltd. Comp Funct Genom 2005; 6: 407-409.
Conference Calendar 409
Proteomics
CHI -- The Protein Engineering Summit
(PEGS), April 24-28
http://www.healthtech.com/2005/pegs/
CHI -- Genomic and Proteomic Sample
Preparation, May 4-5
http://www.healthtech.com/2005/smp/index.asp
54th ASMS Conference on Mass Spectrometry,
May 28-June 1
http://www.asms.org/
Systems Biology
Genomes to Systems Conference, March
22-24
http://www.genomestosystems.org/index.html
1st Maga Circe Conference on Metabolic
Systems Analysis, March 26-29
http://www.assimet.org/circe/pmwiki.php
Transcriptomics/Regulation
CHI -- Quantitative PCR, March 19-21
http://www.healthtech.com/2006/qpc/index.asp
CSHL -- Global Regulation of Gene
Expression, March 23-26
http://meetings.cshl.edu/meetings/systems06.
shtml
KEY -- Regulation of Eukaryotic Transcription:
From Chromatin to mRNA, April 21-26
http://www.keystonesymposia.org/Meetings/
ViewMeetings.cfm?MeetingID=785
CSHL -- 71st CSHL Symposium: Regulatory
RNAs, May 31-June 5
http://meetings.cshl.edu/meetings/symp06.shtml
Biotech Industry
BIO -- Annual International Convention,
March 9-12
http://www.bio.org/events/2006/
BIOnale2006, May 2-4
http://www.bionale.org/
Key
ACS: American Chemical Society: http://www.
acs.org/
ASBMB: American Society for Biochemistry and
Molecular Biology: http://www.asbmb.org/
ASM: American Society for Microbiology: http://
www.asm.org/
BIO: Biotechnology Industry Organization:
http://www.bio.org/
BS: Biochemical Society: http://www.biochemis-
try.org/
BSAS: British Society of Animal Science: http://
www.bsas.org.uk
CHI: Cambridge Healthtech Institute: http://www.
healthtech.com
CSHL: Cold Spring Harbor Laboratories: http://
meetings.cshl.org/index.htm
ESHG: European Society of Human Genetics:
http://www.eshg.org/
EuroSciCon: http://www.euroscicon.com/
GRC: Gordon Research Conferences: http://www.
grc.uri.edu/
HUGO: Human Genome Organisation: http://
www.gene.ucl.ac.uk/hugo/
IASTED: International Association of Science and
Technology for Development: http://www.iasted.
org/
IBC: IBC Conferences: http://www.ibc-lifesci.com
KEY: Keystone Symposia: http://www.keystone-
symposia.org/
NSTI: Nano Science and Technology Institute:
http://www.nsti.org/
SGM: Society for General Microbiology: http://
www.sgm.ac.uk/
The Conference Calendar is a listing of forthcoming conferences of interest to our readership. We provide
the title, dates and web address of each conference, to enable readers to find out more information.
Inclusion of a conference does not imply endorsement by the journal.
Copyright  2006 John Wiley & Sons, Ltd. Comp Funct Genom 2005; 6: 407-409.

				

